# Bradycardia‐Dependent Elevation of Atrial Pacing Threshold Mimicking Lead Failure Early After Pacemaker Implantation

**DOI:** 10.1002/joa3.70478

**Published:** 2026-09-23

**Authors:** Ayano Shibata, Rikitake Kogawa, Yukitoshi Ikeya, Toshiko Nakai, Yasuo Okumura

**Affiliations:** ^1^ Department of Clinical Engineering Itabashi Hospital, Nihon University School of Medicine Tokyo Japan; ^2^ Division of Cardiology, Department of Medicine Nihon University School of Medicine Tokyo Japan

**Keywords:** atrial pacing threshold, pacemaker, phase 4 block, sick sinus syndrome

## Abstract

Atrial pacing failure after pacemaker implantation does not always indicate lead malfunction. Non‐invasive assessment can identify the underlying cause and may avoid unnecessary lead revision.

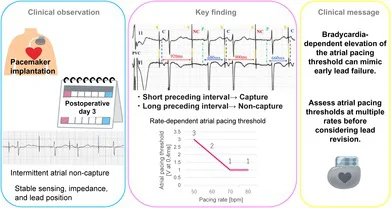

An increase in pacing threshold shortly after pacemaker implantation is a recognized early complication. In many cases, lead‐related problems such as lead dislodgement or micro‐instability are suspected, sometimes leading to early lead revision. However, pacing thresholds may rarely increase in a bradycardia‐dependent manner because of transient changes in myocardial excitability.

A woman in her 70s underwent dual‐chamber pacemaker implantation for sick sinus syndrome. Active‐fixation leads (CapSureFix Novus, Medtronic) were positioned in the right atrial appendage and right ventricular septum. At implantation, the atrial pacing threshold was 1.2 V at 0.4 ms at a pacing rate of 70 bpm. P‐wave amplitude and lead impedance were 1.125 mV and 475 Ω, respectively. The device was programmed in Managed Ventricular Pacing (MVP) mode with a rate range of 60–130 bpm and an atrial output of 3.5 V at 0.4 ms.

On postoperative day 3, atrial pacing failure was observed (Figure 1). Successful atrial capture occurred when pacing stimuli were delivered approximately 660–680 ms after a spontaneous P wave, whereas capture failed when the interval from the preceding atrial activation was prolonged to approximately 900–920 ms. No discernible retrograde P wave was observed following the premature ventricular complex on the surface electrocardiogram. Device interrogation demonstrated an atrial pacing threshold of 0.875 V at 0.4 ms, P‐wave amplitude of 1.3 mV, and lead impedance of 399 Ω. Chest radiography showed no evidence of lead displacement. Because the cause of pacing failure was unclear and intrinsic atrial rhythm was maintained at approximately 50 bpm, the pacemaker was reprogrammed to DDD mode with a lower rate of 50 bpm to prioritize intrinsic rhythm.

**FIGURE 1:**
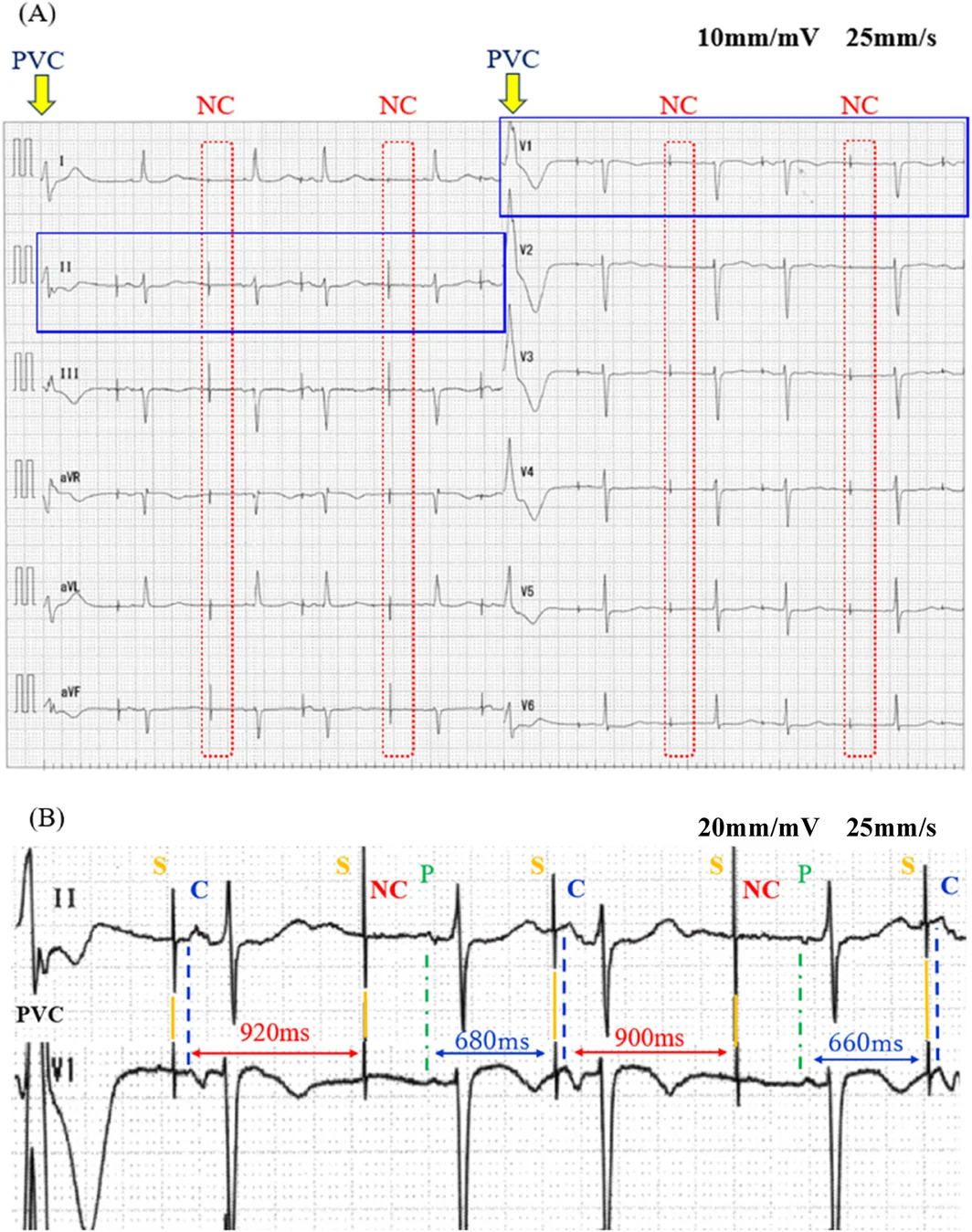
Twelve‐lead electrocardiogram and enlarged tracings demonstrating interval‐dependent atrial capture failure. (A) Twelve‐lead electrocardiogram obtained on postoperative day 3 showing intermittent atrial pacing failure. The boxed region is enlarged in panel B. (B) Enlarged lead II and V1 tracings. Atrial capture occurred when the pacing stimulus was delivered 660–680 ms after the preceding spontaneous P wave, whereas non‐capture occurred after a longer interval of 900–920 ms. No discernible retrograde P wave was observed following the premature ventricular complex. S indicates pacing stimulus; C, capture; NC, non‐capture; and P, spontaneous P wave.

On postoperative day 6, device interrogation suggested an apparent increase in atrial pacing threshold when measured at a pacing rate of 50 bpm in the sitting position, reaching 3.0 V at 0.4 ms. However, the pacing threshold improved as the pacing rate increased: 2.0 V at 60 bpm, 1.0 V at 70 bpm, and 1.0 V at 80 bpm (Figure 2). Lead impedance and P‐wave amplitude remained stable (456 Ω and 1.6 mV, respectively), suggesting that the threshold elevation was not related to lead dysfunction.

**FIGURE 2:**
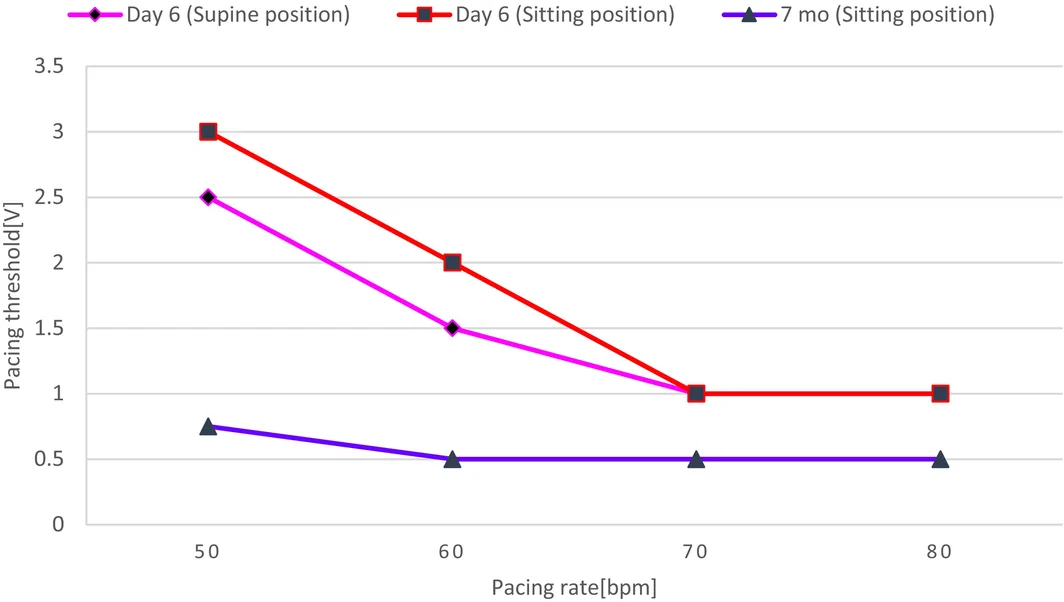
Relationship between pacing rate and atrial pacing threshold. Atrial pacing thresholds were highest at 50 bpm on postoperative day 6 and improved progressively at faster pacing rates, demonstrating a bradycardia‐dependent pattern.

At 1‐month follow‐up, the atrial pacing threshold improved to 0.5 V at 0.4 ms with a P‐wave amplitude of 2.6 mV and lead impedance of 494 Ω. At 7‐month follow‐up, thresholds at pacing rates of 50, 60, 70, and 80 bpm were 0.75, 0.5, 0.5, and 0.5 V at 0.4 ms, respectively, demonstrating resolution of the bradycardia‐dependent threshold elevation. Lead parameters remained stable throughout follow‐up (Figure 3).

**FIGURE 3:**
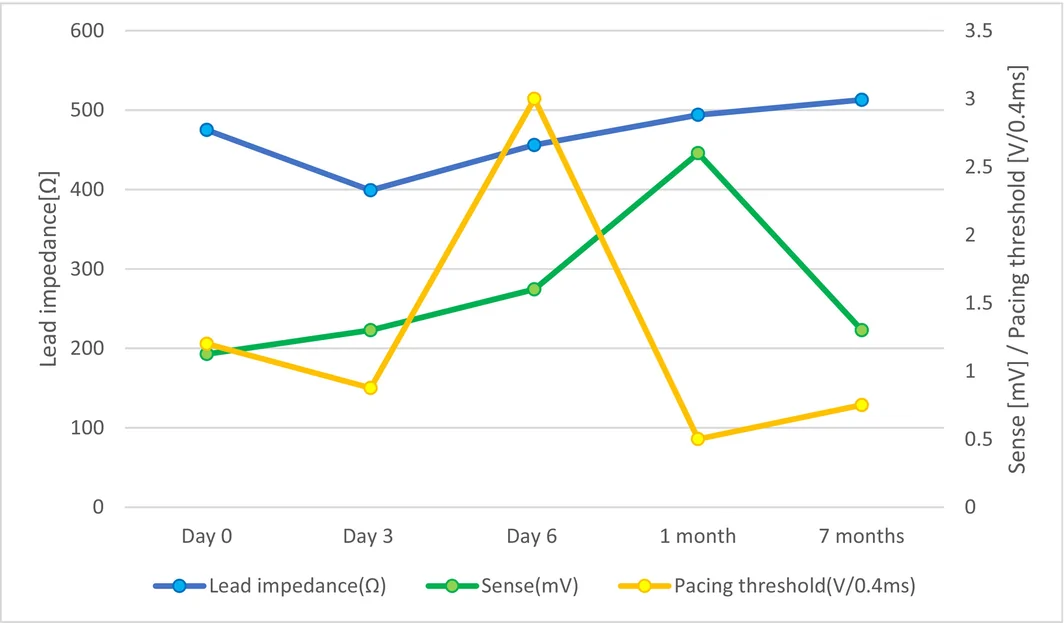
Time course of atrial lead parameters after implantation. Lead impedance and sensing amplitude remained stable, whereas the atrial pacing threshold showed transient bradycardia‐dependent elevation in the early phase and improved during follow‐up.

Bradycardia‐dependent elevation of atrial pacing threshold has been previously reported after pacemaker implantation, particularly in patients with sick sinus syndrome. Kimata et al. described a similar phenomenon in which atrial capture thresholds increased at slower pacing rates and improved at higher rates [1]. Similar rate‐dependent threshold changes have also been reported after leadless pacemaker implantation [2].

The mechanism may be related to phase 4 block, in which partially depolarized myocardial tissue demonstrates reduced excitability at slower heart rates because sodium channels remain inactivated [3]. When the pacing rate increases, recovery of sodium channel availability may restore myocardial excitability and improve pacing thresholds. In the present electrocardiogram, a preceding atrial activation may have transiently modulated myocardial excitability, whereas a longer diastolic interval may have allowed progressive phase 4 depolarization and reduced responsiveness to the pacing stimulus. However, intracardiac atrial electrograms and pharmacological testing with adenosine triphosphate were not performed; therefore, this mechanism could not be definitively demonstrated [4].

Another possible explanation for loss of atrial capture is exit block caused by local myocardial injury at the lead–tissue interface. Exit block may occur transiently after lead implantation because of inflammation or local edema. However, several observations argue against exit block as the primary mechanism in this case. Lead impedance and sensing amplitude remained stable, chest radiography showed no lead displacement, and pacing thresholds consistently improved with increasing pacing rates.

These findings are more compatible with a rate‐dependent electrophysiological mechanism. Nevertheless, subtle changes in the lead tip‐myocardial interface due to micro‐dislodgement cannot be completely excluded despite the absence of radiographically apparent lead migration [5].

The delayed recognition of bradycardia‐dependent pacing failure several days after implantation is also noteworthy. Atrial pacing thresholds were not systematically evaluated at slower pacing rates during the immediate postoperative period, and sufficient output safety margins may have masked rate‐dependent threshold elevation. Thus, the abnormality may have been present but unrecognized earlier, or it may have evolved because of time‐dependent local tissue responses and transient changes in myocardial excitability after lead implantation.

Recognition of this phenomenon is clinically important because bradycardia‐dependent elevation of pacing thresholds may mimic lead failure early after implantation and lead to unnecessary lead revision. In the present case, conservative observation and appropriate device programming allowed spontaneous improvement during follow‐up.

## Funding

The authors have nothing to report.

## Ethics Statement

The research related to human use complied with all relevant national regulations and institutional policies and is in accordance with the tenets of the Helsinki Declaration.

## Consent

The patient has provided consent for publication.

## Conflicts of Interest

Dr. Okumura has received research grants unrelated to this study from Johnson & Johnson KK and Biosense Webster Inc.; scholarship funds from Nippon Boehringer Ingelheim; and speaker honoraria from Daiichi‐Sankyo, AstraZeneca, Bayer Healthcare, Bristol‐Myers Squibb, and Johnson & Johnson KK. He is also affiliated with endowed departments supported by Boston Scientific Japan, Biotronik Japan, Abbott Medical Japan, Japan Lifeline, and Medtronic Japan. The other authors declare no conflicts of interest related to this article.

## Data Availability

The data that support the findings of this study are available from the corresponding author upon reasonable request.
